# Increased activity of MdFRK2, a high-affinity fructokinase, leads to upregulation of sorbitol metabolism and downregulation of sucrose metabolism in apple leaves

**DOI:** 10.1038/s41438-018-0099-x

**Published:** 2018-12-01

**Authors:** Jingjing Yang, Lingcheng Zhu, Weifang Cui, Chen Zhang, Dongxia Li, Baiquan Ma, Lailiang Cheng, Yong-Ling Ruan, Fengwang Ma, Mingjun Li

**Affiliations:** 1 State Key Laboratory of Crop Stress Biology for Arid Areas/Shaanxi Key Laboratory of Apple, College of Horticulture, Northwest A&F University, Yangling, Shaanxi 712100 China; 2 Section of Horticulture, School of Integrative Plant Science, Cornell University, Ithaca, NY 14853 USA; 3 School of Environmental and Life Sciences, The University of Newcastle, Callaghan, NSW 2308 Australia

## Abstract

To investigate the functions of fructokinase (FRK) in apple (*Malus domestica*) carbohydrate metabolism, we cloned the coding sequences of *MdFRK1* and *MdFRK2* from the ‘Royal Gala’ apple. The results showed that *MdFRK2* expression was extremely high in shoot tips and young fruit. Analyses of heterologously expressed proteins revealed that MdFRK2 had a higher affinity for fructose than did MdFRK1, with Km values of 0.1 and 0.62 mM for MdFRK2 and MdFRK1, respectively. The two proteins, however, exhibited similar Vmax values when their activities were significantly inhibited by high concentrations of fructose. *MdFRK2* ectopic expression was associated with a general decrease in fructose concentration in transgenic lines. In leaves, increased FRK activity similarly resulted in reduced concentrations of glucose and sucrose but no alterations in sorbitol concentration. When compared with those in the untransformed control, genes involved in sorbitol synthesis (*A6PR*) and the degradation pathway (*SDH1/2*) were significantly upregulated in transgenic lines, whereas those involved in sucrose synthesis (*SPS1*) and other degradation processes (*SUSY4*, *NINV1/2*, and *HxK2*) were downregulated. The activity of enzymes participating in carbohydrate metabolism was proportional to the level of gene expression. However, the growth performance and photosynthetic efficiency did not differ between the transgenic and wild-type plants. These results provide new genetic evidence to support the view that FRK plays roles in regulating sugar and sorbitol metabolism in *Rosaceae* plants.

## Sugar metabolism: a master regulator

An international study led by Mingjun Li at Northwest A&F University in Shaanxi, China, reveals that an enzyme regulating the utilization of fructose in apple plants, fructokinase 2 (FRK2), has a key role in sugar metabolism. Overexpressing the gene encoding FRK2 in apple plants did not affect their growth or levels of sorbitol, the major product of photosynthesis in these plants, but it significantly lowered the levels of fructose, glucose and sucrose in mature leaves and increased the levels of starch. Accordingly, the activity of enzymes involved in sucrose and glucose metabolism was downregulated, whereas the activity of those involved in sorbitol metabolism was upregulated. Future analysis of the fruit and flowers will shed further light on the role of FRK in controlling sugar levels and the distribution of carbon in apple plants.

## Introduction

Photosynthetically active leaves produce various forms of carbohydrates, which comprise primarily sucrose and starch in most plant species. These compounds serve as energy and carbon sources for metabolism, including the synthesis of cell wall polymers. Soluble sugars—primarily sucrose, glucose, and fructose—can also act as signal molecules to regulate the expression of various genes involved in defense, development, and metabolism^1–4^. Therefore, understanding the mechanisms of carbohydrate metabolism is important for exploiting sugar metabolism to improve plant growth and crop quality.

In the mature green leaves of most plants, sucrose is the main end product of photosynthesis in mesophyll cells^4^. One key enzyme for sucrose synthesis is sucrose-phosphate synthase (SPS; EC 2.4.1.14)^5,6^. Sucrose can be converted to fructose and glucose by invertases (EC 3.2.1.26), including cell wall invertase (CWINV), neutral invertase (NINV), and vacuolar invertase (VINV) or to fructose and UDP-glucose (UDPG) by sucrose synthase (SUSY; EC 2.4.1.13)^4^. The resulting fructose can be phosphorylated by fructokinase (FRK; EC 2.7.1.4) to fructose-6-phosphate (F6P), whereas the resulting glucose can be phosphorylated by hexokinase (HxK; EC 2.7.1.1) to glucose-6-phosphate (G6P)^7–9^. HxK and FRK are central in sugar metabolism and homeostasis.

As outlined above, FRK is the gateway to fructose metabolism, regulating fructose flux in cells^10^. Genes encoding FRK with distinct kinetic properties have been identified from multiple tissues and species, including tomato (*Lycopersicon esculentum*)^11–13^, potato (*Solanum tuberosum*)^14^, maize (*Zea mays*)^15^, rice (*Oryza sativa*)^16^, and *Arabidopsis thaliana*^17^. Generally, FRK2 orthologs have much higher affinity for fructose than do other FRKs^9,17,18^ and play important roles in controlling fructose utilization and carbon flux repartition^19^. In plants that have sucrose as the end product of photosynthesis, scientists have explored the roles of FRK2 orthologs in regulating carbon partition and plant growth via transgenic experiments. The inhibition of the expression of *FRK2* orthologs in transgenic lines of tomato and potato have been found to have negative effects on growth, potato yield, and development of tomato active xylem^20,21^, whereas sugar levels in leaves are largely unaltered^20–22^. However, in transgenic aspen, the reduction of FRK activity by using an RNAi-*FRK2* approach increased the leaf concentrations of fructose, glucose, and sucrose but decreased cell wall fiber thickness and the proportion of cellulose in the cell wall, implying that *FRK2* is required for carbon partitioning to cellulose in the wood^19^. Additionally, overexpression of tomato low-affinity *LeFRK1* in cotton (*Gossypium hirsutum*) results in a decreased level of sucrose in young leaves but increases in seed and fiber yields per plant due to the increased area per leaf and leaf number^23^. Although the physiological roles of *FRK* in plant development have been partly elucidated in these plant species, the underlying function of *FRK* in regulating carbohydrate concentrations remains obscure, and no studies have investigated the impacts of enhanced high-affinity FRK activity on sugar metabolism and plant growth.

Unlike most higher plant species, in which sucrose is the end product of photosynthesis, in apple and many other *Rosaceae* fruit trees, sorbitol is the primary end product of photosynthesis. It is catalyzed via aldose-6-phosphate reductase (A6PR; EC 1.1.1.200) and accounts for 60 to 80% of the newly fixed carbon in source leaves of these trees^24,25^. In addition to being a key metabolite in carbohydrate metabolism, sorbitol acts as a signal that regulates stamen development, pollen tube growth and resistance to *Alternaria alternata* in apple^26,27^. In apple sink cells, almost all the sorbitol available is converted to fructose by sorbitol dehydrogenase (SDH; EC 1.1.1.14), whereas half of the sucrose is converted to fructose^28,29^. As a result, in apple sink cells, more than 80% of the total carbon flux passes through fructose^30^, whereas in other model plant systems that transport and utilize only sucrose (e.g., *Arabidopsis*, tomato, and *Populus*), 50% of the total carbon flux is via fructose^4^. The utility of such high levels of fructose in apple sink cells must require high FRK activity. *MdFRK* expression is thought to be involved in regulating fructose utilization and accumulation in apple^30,31^. In the fruit of transgenic apple with decreased sorbitol synthesis, *MdFRK2* was suggested as a main regulating factor of fructose homeostasis^32^. We hypothesized that apple FRK plays direct roles in metabolizing fructose and controlling its accumulation in apple. Based on analyses of transcript, enzyme activity and metabolite data in different varieties, it was suggested that FRK might play roles in regulating sugar metabolism and concentrations in peach fruits^33^. However, there is no genetic evidence available to clarify whether FRK plays roles in regulating sugar and sorbitol metabolism in *Rosaceae* plants.

Here, we examined the role of *MdFRK2* in regulating carbohydrate metabolism in apple leaves. Its expression patterns and enzymatic properties suggest that *MdFRK2* is the major fructose-phosphorylating gene and that its expression is closely correlated, both temporally and spatially, with fructose metabolism in apple. To investigate its roles in plant growth and sugar metabolism, we generated *MdFRK2*-overexpression (OE) transgenic plants in which the expression and activity of *MdFRK2* was increased significantly relative to that of untransformed wild-type (WT) plants. In the transgenic plants, all fructose, glucose, and sucrose concentrations were decreased; furthermore, the expression of genes and the activities of enzymes involved in sorbitol metabolism were greatly enhanced, whereas those of sucrose metabolism were decreased. The results demonstrate that overexpression of *MdFRK2* in apple alters the allocation of photosynthetic carbon flux from sucrose to sorbitol, thereby changing sugar concentrations and homeostasis in apple leaves.

## Results

### Cloning and expression characterization of *MdFRK2*

**Fig. 1:**
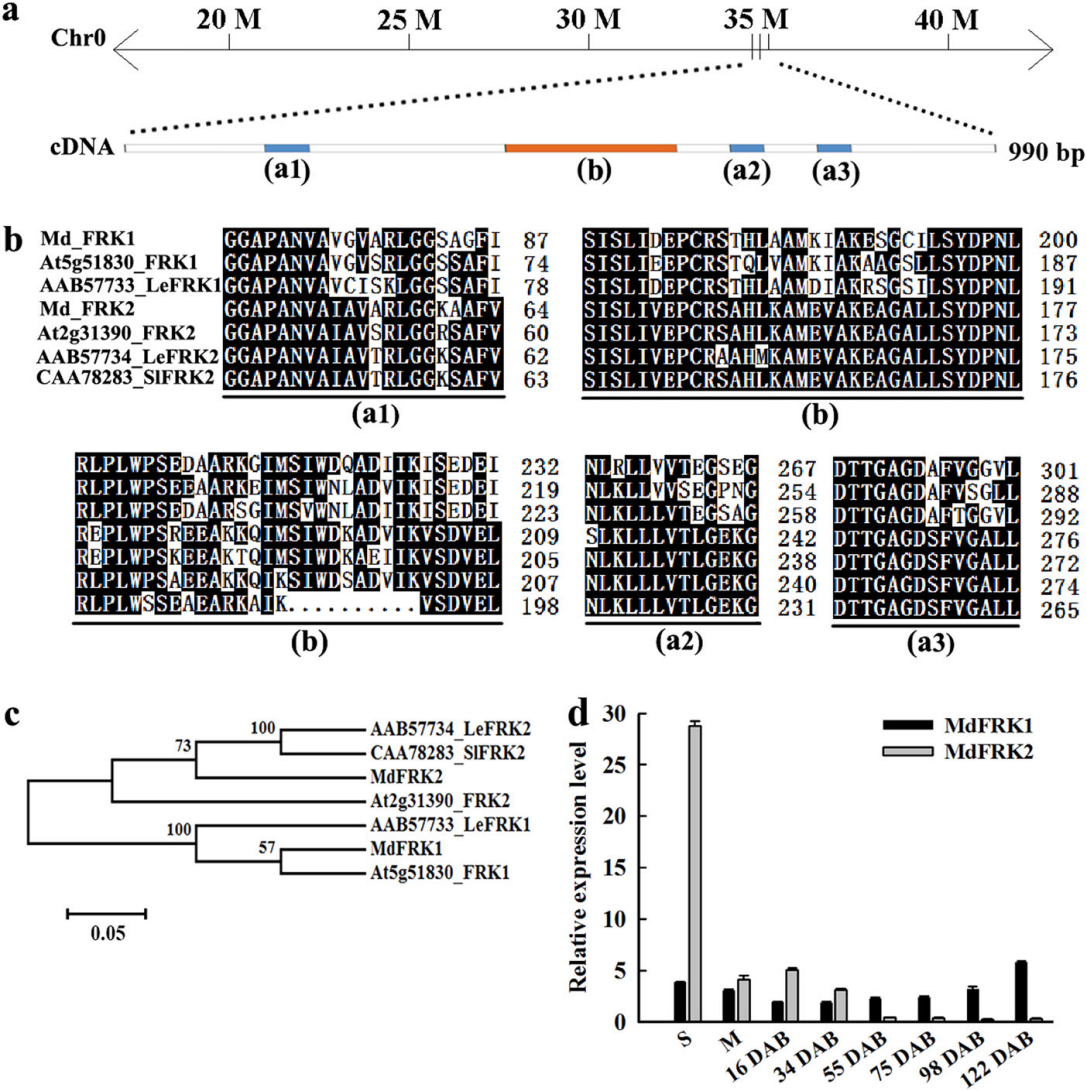
Sequence analysis of *MdFRK1/2*. **a** Schematic diagram of gene positions and structure of *MdFRK2*. Conserved regions are indicated with (a1), (a2), (a3) and (b). **b** Alignment of conserved regions in deduced amino acid sequences of MdFRK1/2 with other plant fructokinases. (a1–a3), 3 signature patterns for phosphofructokinase B (PfkB) family; (**b**), region specific to fructokinase. Black boxes indicate proteins with >50% identical amino acids. **c** Maximum likelihood phylogeny of MdFRK1/2 deduced amino acid sequences and those from *Arabidopsis*, *Lycopersicon esculentum*, and *Solanum tuberosum*. **d** Patterns of mRNA relative expression for *MdFRK1* and *MdFRK2* in developing fruit and different tissues. S, shoot tips; M, mature leaves; DAB, days after bloom

In apple, four *MdFRK* genes are mainly expressed and are homologous to *LeFRK-1* to *-4* from tomato^30^. Our previous studies indicated that *MdFRK2* might be a key gene in determining FRK activity and fructose homeostasis^30–32^. In the present study, to investigate the function of the *MdFRK2* gene, we cloned the coding sequence of *MdFRK2* from ‘Royal Gala’ apple plants, and the *MdFRK1* coding sequence was also cloned as a reference gene. Both genes showed similar sequences as the predicted protein from ‘Golden Delicious’ apple (Fig. 1a). Furthermore, *MdFRK2* showed high amino acid sequence identity to *LeFRK2* (82.4%) and *StFRK2* (79.3%) while *MdFRK1* was homologous to *AtFRK1* and *LeFRK1* (Fig. 1c). Both of the deduced amino acid sequences contained highly conserved motifs and domains of characterized *FRK* genes (Fig. 1b)^10^. In *Arabidopsis* mesophyll protoplasts and onion epidermal cells, we observed that, analogous to tomato *SlFRK1* and *SlFRK2*, both MdFRK1::GFP and MdFRK2::GFP occurred in plasma membrane and cytoplasm, whereas MdFRK2::GFP was also detected in the nucleus (Fig. S1).

We further investigated the expression patterns of *MdFRK1* and *MdFRK2* in different tissues from the ‘Royal Gala’ apple. Our results revealed that *MdFRK1* mRNA was expressed at an approximately constant level in shoot tips (shoot apex meristem with primordia and early leaves including young stem) and mature leaves, whereas *MdFRK2* mRNA levels were approximately six-fold higher in shoot tips than those in mature leaves (Fig. 1d). While the fruits were developing, the transcript levels of *MdFRK1* remained unchanged until just before fruit ripening, when they increased markedly. In contrast, the level of *MdFRK2* mRNA was highest in the earlier stages of fruit development (Fig. 1d).

### Enzymatic properties of MdFRK2

**Fig. 2:**
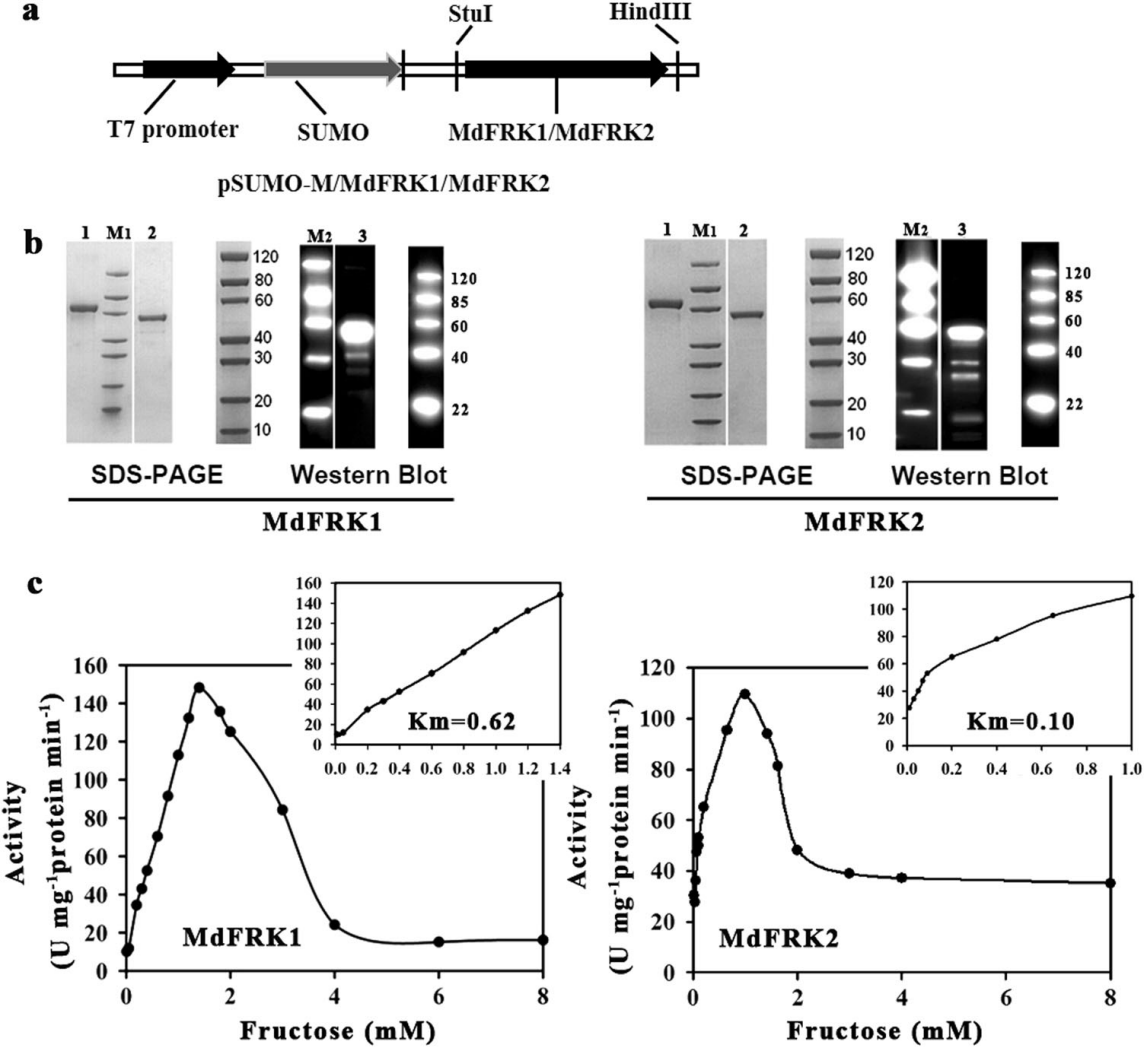
Kinetics analysis of 2 fructokinase isoform proteins from *E. coli* expressing *MdFRK*s. **a** Schematic gene structure of pSUMO-M/MdFRK1 and pSUMO-M/MdFRK2 constructs. **b** SDS-PAGE and western blot results. For MdFRK1 (left): Lanes M1, M2, protein markers; 1, MdFRK1 (2.00 μg); 2, BSA (2.00 μg); 3, FRK1 (His antibody). For MdFRK2 (right): Lanes M1, M2, protein markers; 1, MdFRK2 (2.00 μg); 2, BSA (2.00 μg); 3, FRK2 (His antibody). **c** Effect of fructose concentration on MdFRK1 (left) and MdFRK2 (right) activity in extracts prepared from *E. coli*. Inset shows the level at which fructokinase activity inhibition begins

To examine the catalytic characteristics of the proteins encoded by *MdFRK1* and *MdFRK2*, we constructed pSUMO-M vectors of *MdFRK1* (predicated to be a low-affinity FRK, similar to that in tomato) and *MdFRK2* (Fig. 2a). The two plasmids were individually expressed in *E. coli* with polyhistidine (His) tags and purified using Ni-agarose resin. Analysis using SDS polyacrylamide gel electrophoresis (SDS-PAGE) showed that the molecular weight for each isoform was approximately 60 KD (Fig. 2b). Both of the heterologous proteins had high specificity for fructose but no affinity for glucose (data not shown). Their enzyme activities were significantly inhibited by fructose when the concentration exceeded 2 mM (Fig. 2c). Furthermore, the maximum activity was measured at 1.0 and 1.4 mM fructose for MdFRK2 and MdFRK1, respectively, with Km values of 0.10 mM for MdFRK2 and 0.62 mM for MdFRK1 (Fig. 2c). The results indicate that MdFRK2 has much higher affinity for fructose than does MdFRK1 and a lower Vmax for fructose.

### Overexpression of *MdFRK2* increased FRK enzyme activity in leaves

**Fig. 3:**
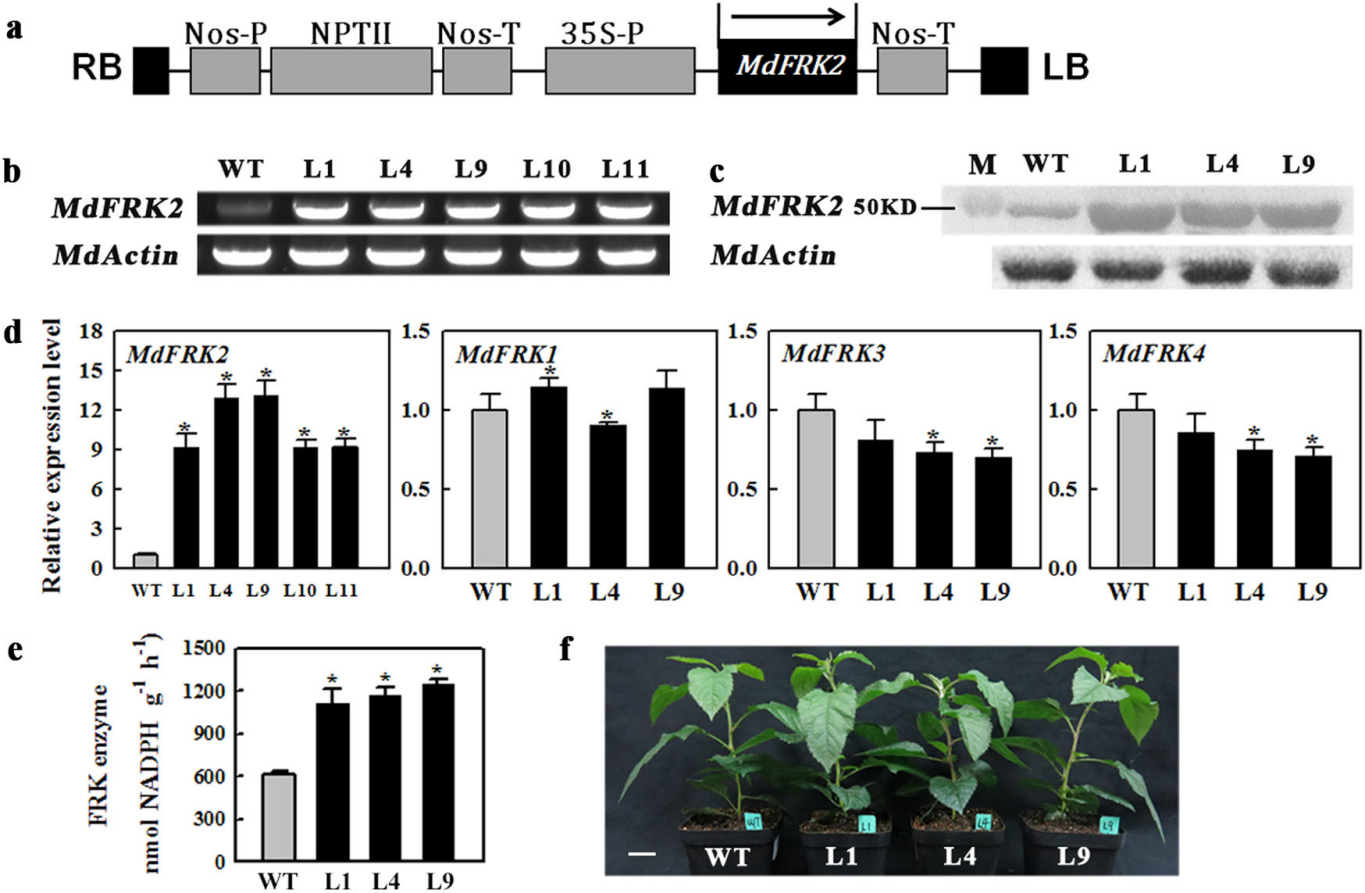
Levels of transcripts, enzyme activities, and MdFRK2 protein in leaves from transgenic lines. **a** Schematic diagram of vector construct. **b** Semiquantitative RT-PCR. WT, wild-type; L1, L4, and L9, *MdFRK2*-OE lines. **c** Western blotting using the specific monoclonal antibody generated from rabbit. Amount of protein extract loading was referenced by immunoblot analysis with anti-Actin antibody. M: protein marker. **d**, **e** Quantitative RT-PCR of *MdFRKs* expression and MdFRK activity in mature leaves from wild-type (WT) and transgenic lines (L1, L4, and L9), respectively. **f** Phenotype of wild-type (WT) and transgenic lines (L1, L4, and L9). In (**b**, **d**), the *MdActin* gene was used as internal control. Error bars represent SD based on 3 independent replicates. Statistically significant differences among values (at *P* < 0.05) were detected with independent *t*-tests and SPSS software. Scale bar = 2 cm

In an attempt to determine the potential physiological functions of *MdFRK2* in apple, we tried to generate *MdFRK2-*inhibited lines using RNAi technology. However, the buds from the transgenic seedlings did not grow well and eventually died in the selection medium, possibly because the gene is indispensable for the growth of the shoot apical meristem, as indicated by its high expression in the shoot tip (Fig. 1d). As an alternative, we constructed an overexpression cassette with its open reading frame (ORF) inserted under the control of a 35 S promoter and transformed it into apple (Fig. 3a). We identified five overexpression transgenic lines through RT-PCR (Fig. 3b) and qRT-PCR (Fig. 3d), which exhibited evident increases in *MdFRK2* transcript levels in mature leaves relative to the levels in WT controls (Fig. 3b, d). Among these lines, L1, L4, and L9 displayed 9.2, 13.0 and 13.2-fold increases, respectively, in leaf mRNA levels, respectively, when compared with that of relative to the levels of the untransformed WT controls (Fig. 3d). All three lines also exhibited protein abundance using specific antibody (Fig. 3c) and significantly increased enzyme activity (Fig. 3e). For the other *FRK* members, the expression of *MdFRK1* did not differ from that of WT controls, whereas the transcript levels of *MdFRK3* and *MdFRK4* were slightly decreased, indicating that any phenotype observed in the transgenic plants would be largely due to the overexpression of *MdFRK2*.

### Overexpression of *MdFRK2* altered carbohydrate levels in leaves

**Fig. 4:**
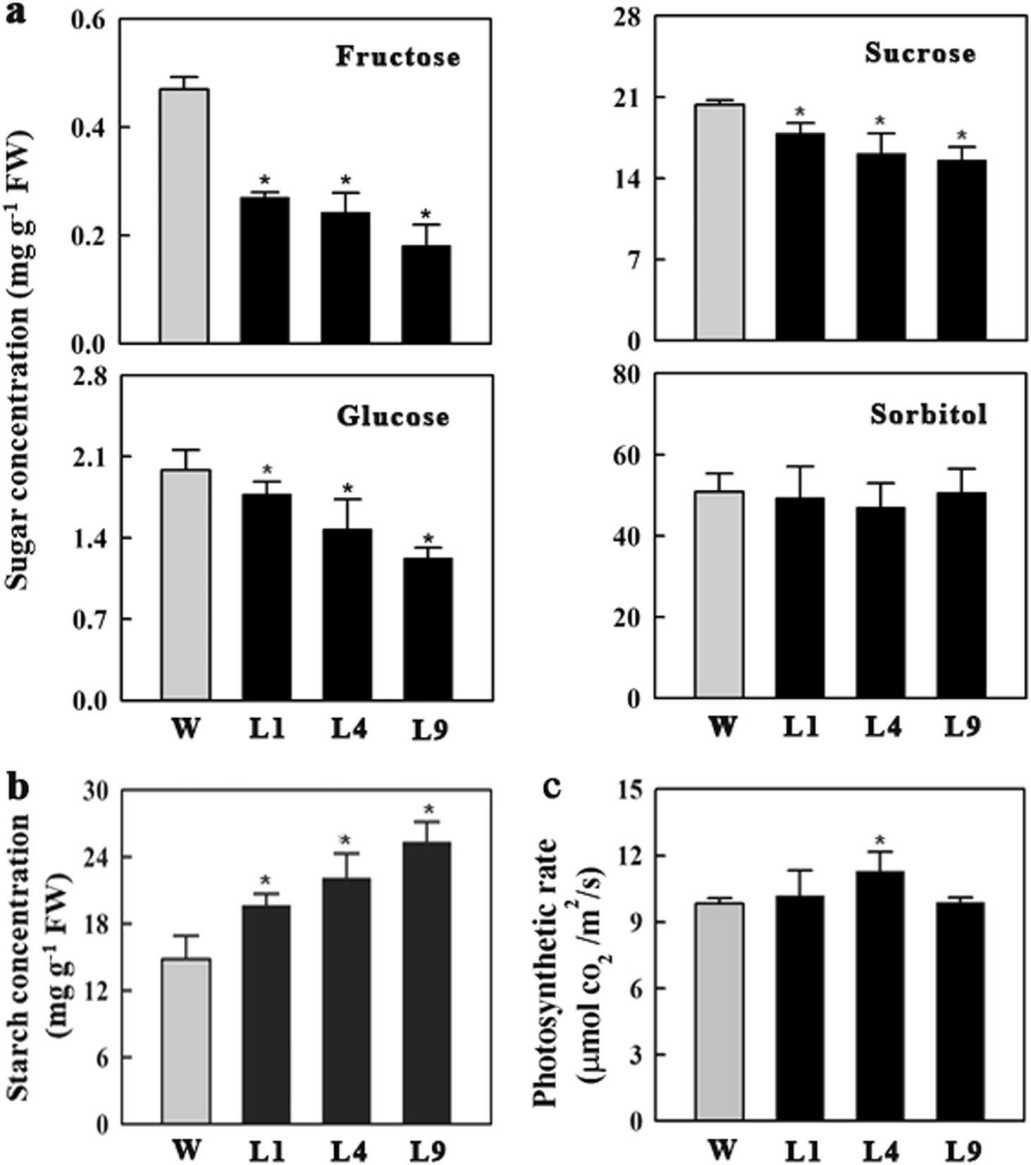
Changes in carbohydrate concentrations and photosynthetic rates in mature leaves of wild-type (WT) and transgenic lines (L1, L4, and L9). **a** Concentrations of sorbitol, sucrose, glucose, and fructose. **b** Starch concentrations. **c** Photosynthetic rates. Error bars represent SD based on 3 independent replicates. Statistically significant differences among values (at *P* < 0.05) were detected with independent *t*-tests and SPSS software

Increased MdFRK activity in the leaves resulted in significantly lower concentrations of fructose in the transgenic lines, with levels reduced to 52% of the control level for L1, 50% for L4, and 41% for L9 (Fig. 4a). In the transgenic lines, the concentrations of sucrose and glucose were also decreased (Fig. 4a). By contrast, the level of starch was increased greatly in all three OE lines relative to the level in WT (Fig. 4b). However, the level of sorbitol did not differ between the transgenic and wild-type plants. These data demonstrated that *MdFRK2* modulates sugar concentrations in the leaves; not only those of fructose but also those of glucose and sucrose. In addition, regardless of these observed differences, the transgenic plants showed no obvious alterations in their growth performance or photosynthetic efficiency (Fig. 3f; Fig. 4c; Table S1)

### Overexpression of *MdFRK2* altered the activities of enzymes involved in carbohydrate metabolism

**Fig. 5:**
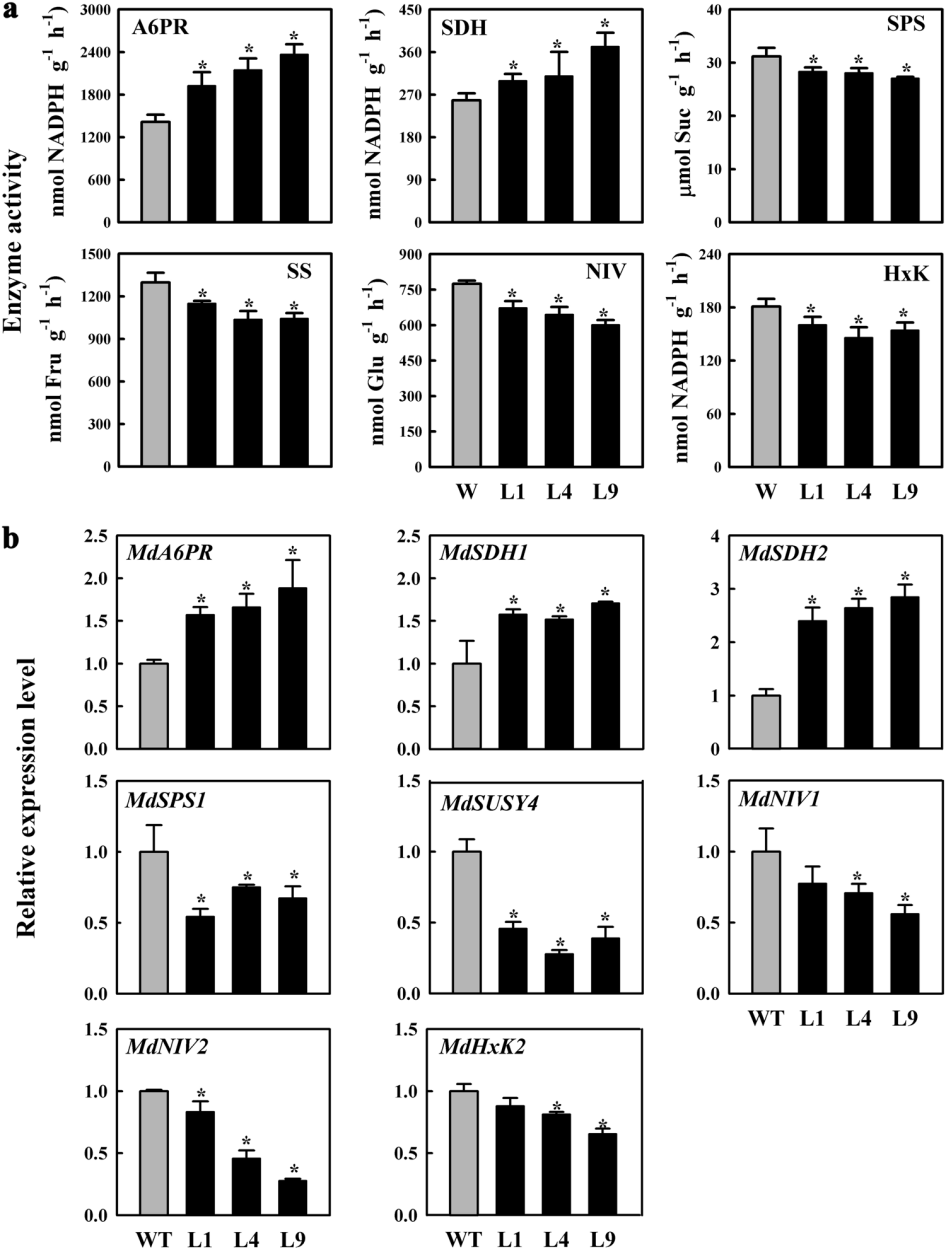
Changes in enzyme activity and gene expression in mature leaves. Activities of enzymes (**a**) and relative mRNA expression of genes (**b**) related to sucrose and sorbitol metabolism in mature leaves of wild-type (WT) and transgenic lines (L1, L4, and L9). Quantitative RT-PCR was performed with gene-specific primers using *MdActin* as an internal control. Error bars represent SD based on 3 independent replicates. Statistically significant differences among values (at *P* < 0.05) were detected with independent *t*-tests and SPSS software

To determine why glucose and sucrose concentrations were reduced in the leaves of *MdFRK2*-overexpression transgenic plants, we analyzed the key enzymes involved in apple soluble sugar metabolism (Fig. 5a). The activities of A6PR and SDH were markedly increased in the OE lines (Fig. 5a). However, key enzymes involved in sucrose metabolism were suppressed to varying degrees. For example, SPS activity was decreased to 77 and 75% of the WT level for L4 and L9, respectively. A similar pattern was observed for SUSY, NIV, and HxK activities. Changes in the status of sucrose and sorbitol (Fig. 4a) agreed with the patterns of enzyme activities involved in sucrose and sorbitol metabolism (Fig. 5a). We also detected the expression levels of main genes involved in sugar metabolism (Fig. 5b, S3). Notably, genes involved in sorbitol synthesis (*A6PR*) and the degradation pathway (*SDH1* and *SDH2*) were significantly upregulated in the transgenic lines (Fig. 5b). In contrast, the expression of several genes responsible for sucrose synthesis (*SPS1*) and degradation (*SUSY4*, *NINV1*, and *NINV2*) was downregulated in these lines. Corresponding to the reduced glucose concentrations (Fig. 4a), HxK members such as *HxK2* were also slightly suppressed. However, the expression levels of the genes *MdSPS6*, *MdSUSY1/2/3*, *MdNIV3*, *MdHxK1/3/6* were unchanged relative to control levels in transgenic apple leaves (Fig. S3).

## Discussion

### *MdFRK2* is a major player in fructose metabolism in apple

Fructose metabolism in apple exhibits a well-defined developmental pattern. Total FRK activity, as well as fructose levels in different tissues and at various fruit development stages, has been reported previously^30^. The early stage of fruit development was characterized by high FRK activity and low levels of fructose accumulation. FRK expression and activity decrease significantly as the concentrations of fructose peaks in mature fruit^30^. Orthologs of *FRK2* that are mainly located in cytoplasm have been confirmed as the major fructose-phosphorylating enzymes in several species, and they show high expression/abundance in sink tissues^11,15,16,20,34^. Both *MdFRK1* and *MdFRK2* were mainly located in cytoplasm (Fig. S1). The results indicate that the two encoding proteins are involved in fructose phosphorylation in cytoplasm. *MdFRK2* was also expressed in the nucleus, and this expression implies that it plays a role in sugar phosphorylation in the nucleus or has other unknown functions. *MdFRK2* had significantly high expression levels in the shoot tips and in young developing fruit, whereas the levels were substantially lower in mature fruit (Fig. 1d). The expression pattern of *MdFRK2* closely matches the activity of FRK (Fig. 1d)^30^. In contrast, *MdFRK1* showed significantly different tissue-specific and developmental expression patterns from those of *MdFRK2* (Fig. 1d). Additionally, the *MdFRK2-*OE lines exhibited significantly increased enzyme activities and decreased relative fructose levels in mature leaves. (Figs. 3e, 4a). These findings suggest that *MdFRK2* is a major contributor to fructose metabolism in apple; as suggested in peach, high-affinity FRK could be responsible for the low fructose-to-glucose ratio phenotype^33^.

Sorbitol accounts for approximately 60–80% of all photosynthates produced in apple leaves^25^. Almost all of the sorbitol and half of the sucrose are converted to fructose in sink organs. Therefore, at least 80% of the total carbon flux is estimated to pass through fructose in apple sink organs^30^. We expect those organs that do not accumulate significant amounts of fructose (i.e., shoot tips and young fruit) to have stronger FRK activity for fructose utilization than those organs that actively accumulate fructose (e.g., in fruit during cell expansion). Two characteristics of *MdFRK* support this idea. First, *MdFRK2* was more highly expressed in tissues without fructose accumulation, such as shoot tips and young fruit, than in those of mature fruit (Fig. 1d). Second, and more importantly, our results indicated that apple *MdFRK2* purified from *E*. *coli* had a Km value for fructose of ~ 100 µm, whereas the corresponding value for *MdFRK1* was ~ 620 µm (Fig. 2c). The Km value indicated that MdFRK2 has a much higher affinity for fructose than does MdFRK1. However, the maximum fructokinase activity was lower for *MdFRK2* (~ 110 U) than for *MdFRK1* (~ 150 U) (Fig. 2c). This finding is in contrast to findings for tomato, in which the maximum enzyme activity for LeFRK2 protein was less than 10% of that measured for LeFRK1^11^. The high catalytic capability of *MdFRK2* is related to its great capacity for fructose phosphorylation in apple sink cells due to high fructose flux. These results further support our conclusion that *MdFRK2* plays a key role in regulating fructose metabolism in apple.

Similar to the LeFRK2 enzymes^11^, the MdFRK1 and MdFRK2 enzymes in vitro showed substrate inhibition by fructose at pH 8.0. This MdFRK substrate inhibition implied that FRK plays an important gateway role in sugar metabolism and in determining fructose concentrations in apple. This inhibition can effectively control carbon flux into glycolysis and other pathways via F6P once excess fructose exists in the cytosol (especially in sink cells) given that excess F6P can lead to a stress reaction because the cell requirement is limited for carbon flux^4^. In apple, this substrate inhibition is particularly important because the sink cells have much greater fructose carbon flux. This substrate inhibition induces serial accumulations of fructose in the cytosol^10^, and these accumulations then accelerate the storage of fructose in the vacuoles. This outcome suggests that FRK and SUSY work in tandem to control the utilization of sucrose, as is also evidenced by the elevated fructose levels in tomato fruit^9^.

### Increased fructokinase activity alters sugar metabolism in apple leaves

The significant increase in FRK enzyme activity in apple plants that overexpressed *MdFRK2* further confirmed that the *MdFRK2* gene has a decisive role in controlling fructose phosphorylation. As we had expected, elevated FRK activity significantly decreased the fructose concentrations in mature leaves, similar to the connection between diminished FRK expression and higher fructose levels in potato^21^ and aspen^19^. Transgenic cotton plants that overexpressed tomato *LeFRK1* have significantly increased FRK enzyme activity; however, fructose concentrations are unchanged in the leaves of homozygous T3 plants^23^. Km values are higher for *LeFRK1* (1300 µm) than those for *LeFRK2* (54–220 µm), and almost no enzyme activity occurs under low-fructose conditions in yeast systems^11^. Given the enzymatic property of *LeFRK1*, the lack of change in fructose levels when tomato *LeFRK1* is overexpressed in cotton can be attributed to its low affinity^23^. We also confirmed, based on the reduced fructose concentrations, that *MdFRK2* protein has higher affinity for fructose than does *MdFRK1* protein.

In plant cells, fructose is a signal molecule for regulating growth and development^35^. A system that controls fructose levels might help avoid the negative effects associated with fluctuations in fructose concentration. In transgenic lines of tomato and potato, fructose levels are largely unaltered after FRK activities are modulated^21,22,36^. Because they produce sorbitol, apple plants have at least two alternative approaches for compensating for reduced fructose concentrations in cells. These approaches include the conversion of sorbitol to fructose by SDH and a sucrose-cleavage pathway via SUSY or invertase catalysis. We found that leaves from our transgenic apple had significantly increased SDH expression and activity but reduced levels of SUSY and invertase relative to controls (Fig. 5). *MdFRK2* transcription was also strongly increased by exogenous sorbitol feeding than that by sucrose feeding (Fig. S4), confirming that sorbitol is a more versatile substrate than sucrose for fructose metabolism, as reported in peach by Desnoues et al. (2018)^33^. Our results indicated that the decreased fructose concentrations in the leaves were partly compensated by increased sorbitol dehydrogenization via SDH in the *MdFRK2*-OE lines.

When sorbitol synthesis is downregulated in the leaves of transgenic lines, the decreased content of sorbitol leads to a decline in SDH activity and expression level in the shoot tips^29^ and fruit^37^. Sorbitol availability is also thought to affect SDH activity and fructose concentrations in apple fruit^38^. Accordingly, although the ratio of sorbitol/sucrose was increased (Fig. 4a) along with a significant rise in transcript levels and A6PR activity in the transgenics (Fig. 5), sorbitol levels did not differ from control levels because of increased SDH activity in our *MdFRK2*-OE lines.

**Fig. 6:**
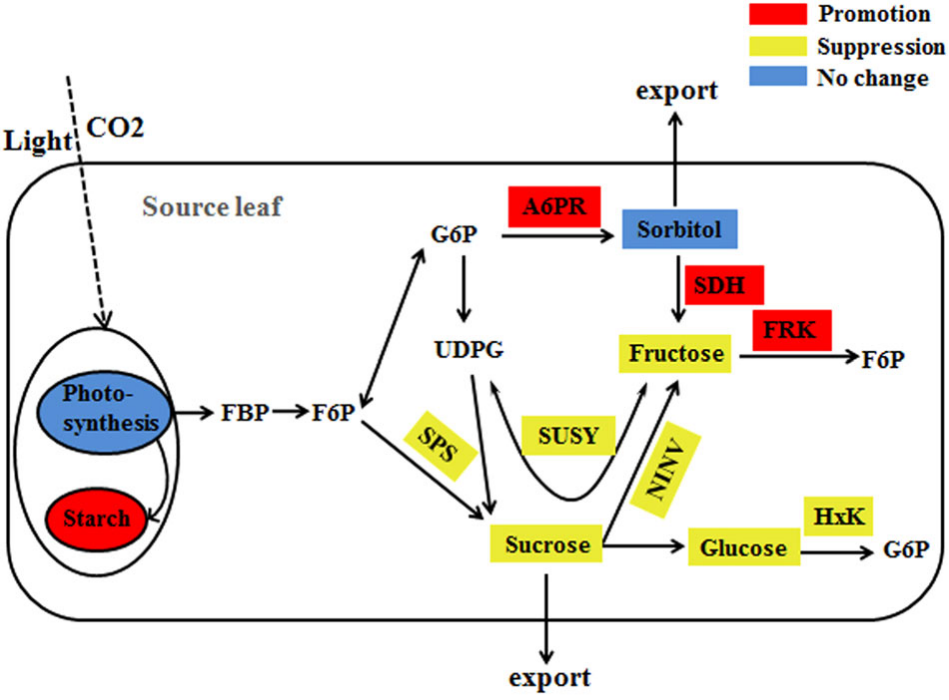
Alteration of pathways for sucrose and sorbitol metabolism in leaves from transgenic apple. Increased FRK activity contributes to the upregulation of sorbitol metabolism (synthesis and dehydrogenation) and downregulation of sucrose metabolism (synthesis and disassembly). Red boxes indicate increases in sugar concentrations or enzyme activity; yellow boxes, reduction in sugar concentrations or enzyme activity; blue boxes, no change in photosynthetic rate or sugar concentrations. Bold lines indicate that metabolism is accelerated

Although the capacity to fix CO_2_ in the leaves was unchanged in OE lines relative to controls (Fig. 4c, Table S1), sugar metabolism and accumulation were significantly changed from control levels in OE lines. *SPS1* is thought to be the main gene responsible for SPS activity^30,39^. Accordingly, decreased *SPS1* expression led to reduced sucrose concentrations. In apple shoot tips, the expression levels and enzyme activities of SUSY and neutral invertase are positively regulated by sucrose concentration^29^, and SUSY activity is correlated with the sink strength of the storage organs^40^. We speculate that the decreases in sucrose synthesis and concentrations reduced SUSY and NIVN activities in *MdFRK2*-OE leaves and downregulated the expression levels of *MdSUSY4* and *MdNINV1* (Fig. 5), both of which are expressed in apple leaves^30^. The decreases in HxK activity and transcript levels of *MdHxK2* (Fig. 5) in the transgenics indicate that *MdHxK2* contributes to HXK activity^7^. Based on these changes in sugar metabolism, we conclude that via a regulated pathway, overexpression of *MdFRK2* leads to declines in the concentrations of fructose, sucrose, and glucose (Fig. 6).

Although we did not detect the level of F6P or G6P, we expected to see an increase in F6P flux because of elevated fructose phosphorylation in the *MdFRK2-*OE lines. Such an increase would mean more F6P being reversibly converted to G6P or fructose-1,6-bisphosphatase^41^. As part of a feedback response, such changes in carbon flux may contribute to greater fixing of CO_2_ for starch synthesis in the daytime; however, the transgenic samples contained significantly more starch in mature leaves (Fig. 4b). It appears that in sink tissues, *FRK* regulates starch metabolism based on the concomitant activities of fructokinase, *SUSY*, and starch-related genes^42^. However, silencing of *LeFRK1* and *LeFRK2* in tomato and *StFRK1* in potato does not alter the patterns of starch accumulation in those species^21,36^. Therefore, the mechanism that links *FRK* with regulated starch concentrations might be associated with its capacity to control sugar metabolism and carbon distribution.

In summary, *MdFRK2* encodes a high-affinity fructokinase, and its overexpression increases fructose phosphorylation and alters carbohydrate metabolism in apple leaves. In apple sink cells, at least 80% of the total carbon goes primarily through fructose into metabolism flux because of sorbitol^30^. Accordingly, apple contains high-affinity FRK that utilizes and maintains fructose at a low level in the cytosol. Here, we confirmed that compared with other plant *FRK2*s, *MdFRK2* is highly expressed in apple sink tissues, and its protein not only shows high affinity for fructose but also has high maximum enzymatic activity. As a main gateway to carbon metabolism, MdFRK activity is strongly inhibited by high fructose concentrations. As in the cases of tomato^20^ and aspen^19^, *MdFRK2* might be required for apple plant development and growth. This outcome might explain why we were unable to obtain *MdFRK2-*silencing lines under the 35S promoter. As an alternative approach, it may be feasible to use an inducible promoter with *MdFRK2*-silencing for apple cultivars that are difficult to transform. For *MdFRK2*-OE plants, the decrease in fructose level was partly compensated by an increase in sorbitol synthesis and a reduction in sucrose synthesis in the leaves. This outcome caused the concentrations of fructose, sucrose, and glucose to decline in the leaves of our transgenics. The molecular mechanism by which sugar metabolism is regulated through a fructose signal is unknown; it is unknown how the signal is perceived and how its homeostasis is regulated via altered metabolism and other pathways. There have been previous reports of fructose homeostasis in apple fruits under different crop loadings^24^, fruits from transgenic apple plants with inhibited sorbitol synthesis^37^, and aspen leaves that show increased CWINV activity^43^. The lack of change in sorbitol level in our transgenic apple showed that this sugar has primary roles in the vegetative growth of *Rosaceae* fruit trees^37,44^. Furthermore, we did not see any phenotypic differences among genotypes, wild or transformed, even though these plants had been grown in the greenhouse for two years. We expect the transgenic apple trees to flower and fruit after four to six years, at which time we will further investigate fruit traits.

## Materials and methods

### Plant materials and growth conditions

Plants of *Arabidopsis thaliana* (‘Columbia’, ecotype Col-0) were maintained in a controlled culture room at 22 °C under conditions of 100 μmol photons m^−2^ s^−1^, 70% relative humidity, and a 16-h, long-day photoperiod. Tobacco plants (*Nicotiana tabacum* L., ‘Samsun NN’) were grown at 25 °C under a 16-h photoperiod and lamp-supplemented light at 120 μmol photons m ^−2^ s ^−1^.

Tissue-cultured plants of WT and *MdFRK2*-transformed ‘Royal Gala’ apples were initially grown on MS medium supplemented with 0.2 mg L^−1^ IAA and 0.3 mg L^−1^ 6-BA for four weeks. They were then transferred to rooting medium (MS + 0.5 mg IBA and 0.5 mg IAA). After rooting, plants of both genotypes were transferred to a culture room maintained at 23 °C under a 14-h photoperiod supplemented with fluorescent light (60 μmol m^−2^ s^−1^)^45^. After these plants had grown for two months, the fifth to eighth leaves from the base of the stem (fully mature leaves) were removed from healthy, uniformly sized stocks. The samples were immediately frozen in liquid nitrogen and stored at –80 °C for further analysis.

### Gene cloning, phylogenetic analysis, and subcellular localization

The predicted sequences of *MdFRK1/2* (MDP0000173131 and MDP0000323311) in the apple genome were retrieved from the *Malus* Genome Database (http://www.rosaceae.org), and primers were designed for gene cloning (Table S2). Total RNA was extracted from apple shoot tips by the CTAB method^46^, and the full cDNAs of *MdFRK1/2* were cloned by RT-PCR.

Amino acid alignments were aligned using ClustalW and Lasergene software (DNASTAR, USA). We then constructed maximum likelihood (ML) phylogenetic trees with 100 bootstrap replicates with MEGA version 5 (http://www.megasoftware.net/). The evolutionary history was inferred according to the ML method based on the JTT matrix-based model^30^.

For determining subcellular localizations, the full-length ORFs of *MdFRK1* and *MdFRK2* without the stop codons were cloned into PBI121-GFP vectors under the control of the CaMV35S promoter. To examine transient expression, we transformed the plasmids into onion epidermal cells with a biolistic helium gun device (PDS-1000; BioRad, Hercules, CA, USA). Cell culturing and fluorescence observations were conducted as previously described^47^. These constructs were also transiently expressed in *Arabidopsis* protoplasts via polyethylene glycol-mediated transformation based on a protocol previously described^48^. After 12 h of transformation, protoplasts were observed with confocal microscopy (LSM 510 META, Carl Zeiss, Germany).

### Expression of *MdFRK*s in *E*. *coli*, protein purification, and kinetics analysis

The expression of *MdFRK*s was monitored in *E*. *coli*, and proteins were purified according to published protocols^49^. After cloning, *MdFRK1* and *MdFRK2* were inserted into pSUMO-M vector. The two recombinant plasmids, pSUMO-M/*MdFRK1*, and pSUMO-M/*MdFRK2*, were then transferred into *E. coli* BL21 (DE3), and the recombinant strains were induced by 1 mM isopropyl–D thiogalactopyranoside (IPTG) to produce fusion proteins tagged with His at 15 °C or 37 °C for different durations. The expressed fusion proteins were detected by SDS-PAGE and western blotting (Fig. S2). After determining that expression peaked at 4 h at 37 °C, we obtained the protein from the supernatant and purified it on 5 × 5 mL HisTrap HP columns prepacked with Ni Sepharose (GE healthcare, Beijing, China) to obtain soluble protein.

For the Km and substrate-specific analyses, fructose phosphorylation activity was measured in a final volume of 0.5 mL that contained 50 mM Tri-HCl (pH 8.0), 4 mM MgCl_2_, 2.5 mM ATP, 0.33 mM NAD^+^, 1 U of G6P dehydrogenase, and 1 U of phosphoglucoisomerase^30^. Fructose concentrations ranged from 1 to 5 mM, whereas glucose concentrations ranged from 0 to 20 mM. Both sugars were measured using a continuous assay that coupled fructose phosphorylation to NADP^+^ reduction at 340 nm.

### Vector constructs and apple transformation

To construct the vector for the MdFRK2-overexpression lines, we introduced the coding region of *MdFRK2* into the pGWB401 binary vector, which is driven by the CaMV 35S promoter and carries the kanamycin (Kan) selectable marker. The recombinant plasmid was introduced into *Agrobacterium tumefaciens* strain EHA105. Leaf fragments of the ‘Royal Gala’ apple were transformed based on a protocol previously described^50^. The regenerated Kan-resistant buds were screened on an MS medium containing 25 mg L^−1^ Kan as a selectable marker. Afterward, plants displaying normal growth were evaluated by PCR analysis of extracted DNA. Overexpression of *MdFRK2* was confirmed by quantitative real-time PCR and western blotting. From the five independently transformed lines obtained here, we selected three for further analysis. Untransformed ‘Royal Gala’ plants were cultured in the same way and served as the control plants.

### Western blotting

Total protein samples were extracted from the mature leaves of OE-lines and WT plants as described previously^45^, and the total concentrations were determined with protein assay kits (Bio-Rad), using bovine serum albumin as a standard. Specific monoclonal antibodies to a peptide (CNPSADMLLKPDELN) from a highly conserved region in MdFRK2 (Genscript, Nanjing, China) were generated in rabbit. MdActin was monitored with a monoclonal antibody (CWBIO, Beijing, China). The procedures for western blotting followed Sun et al. (2017)^45^. The antigen–antibody complexes were detected using Clarity^TM^ Western ECL Substrate (Bio-Rad) according to the manufacturer’s instructions.

### Measurements of soluble sugars and starch

As we described previously^30^, soluble sugars were extracted and derivatized sequentially with methoxyamine hydrochloride and N-methyl-N-trimethylsilyl-trifluoroacetamide. Then, the metabolites were analyzed with a Shimadzu GCMS-2010 SE (Shimadzu Corporation, Kyoto, Japan). The tissue residue that remained after 75% methanol extraction for GC‒MS analysis was re-extracted three times with 80% (v/v) ethanol at 80 °C, and the pellet was retained for starch determinations^30^. The assimilation of CO_2_ in seedling leaves was monitored between 9:30 and 11:30 A.M. using a LI-COR 6400 portable photosynthesis system (LI-COR, Huntington Beach, CA, USA).

### Assays of enzyme activities

We applied methods previously reported methods^30^ to assay the activities of FRK, SDH, NINV, SUSY, HK, and SPS in leaf samples from five biological replications^30^. The A6PR enzyme was extracted and assayed according to the method of Cheng et al. (2005)^25^.

### Analysis of mRNA expression

Quantitative reverse transcription-polymerase chain reaction (qRT-PCR) was used to analyze the expression of genes involved in sugar metabolism. All of the genes, primers, and procedures used here were described previously^30^. qRT-PCR was performed on an ABI7300 Real-Time PCR System (Thermo Fisher Scientific). Transcripts of *Actin* (CN938023) served to standardize the cDNA from our test genes. For each sample, total RNA was extracted from three biological replicates before the qRT-PCR experiments were conducted. All data were examined according to the ddCT method.

### Statistical analysis

All data were analyzed via IBM SPSS Statistics 21 and graphed with Sigma Plot 10.0 software. Data were analyzed using independent *t*-tests with significance evaluated at *P* < 0.05. Values were presented as the means ± standard deviation of 6 plants, with triplicate observations for each measurement.

## Electronic supplementary material

Supplementary material
