# Supplementary material for: Increased activity of MdFRK2, a high-affinity fructokinase, leads to upregulation of sorbitol metabolism and downregulation of sucrose metabolism in apple leaves
Source: Hortic Res. 2018 Dec 1;5:71. doi: 10.1038/s41438-018-0099-x (PMC6269498; doi:10.1038/s41438-018-0099-x)
Supplement: Supplementary file 1 — Supplementary material [file 41438_2018_99_MOESM1_ESM.docx]

**Supplementary Table 1** Height and diameter is shown SE of WT and transgenic lines.

**Diameter at 10cm above soil (mm)**

**Height (cm)**

**Number of trees**

**Genotype**

**3 ±0.4**

**4 ±0.4**

**13±3**

**15±4**

**11±4**

**8**

**9**

**10**

**3 ±0.3**

**14 ±2**

**4 ±0.2**

**10**

**L9**

**WT**

**L1**

**L4**

Heights and diameters of wild-type (WT) and transgenic lines (L1, L4, and L9). Differences among genotypes were not significant at *P <0.05*, based on Student’s *t*-tests.

**Supplementary Table 2** Primers used in this study.

| **Accession no.** | **Sequence (5’-3’)** | **usage of primers** |
| --- | --- | --- |
| MdFRK2 | F:CCAACTTCCATCATTACACGCAGC | Vector construction for apple plant transformation |
|  | R:CCATCCCACCAAAACTCTGTTCCT |  |
| MdFRK1dw | F:CGGTCGACGCCATGGCTAATAATCTC | subcellular localization |
|  | R:AGCACTAGTTTTTTCTCAGCAGCCTGCT |  |
| MdFRK2dw | F:CGGTGACGTTATGGCTAACAATGGCG | subcellular localization |
|  | R:ATCACTAGTTTTGCTTTGTAGAGGGTGA |  |
| MdFRK2P | F:CTGTTTAGTCATCAATTAAGATC | Vector construction for *Arabidopsis* and tobacco plant transformation |
|  | R:TTCACTAATGGATTATTTATGTC |  |
| qMdA6PR | F: GCGCGACTCTCTCGTCAAA | Quantitative expression of MdA6PR |
|  | R:CCAAAGGTGAAACAGAACCAAAC |  |
| qMdSDH1 | F:CTGTACCAGAGGCACCTCCGAGT | Quantitative expression of MdSDH1 |
|  | R:TTGCATGCTCTGCTCTCTCGTTG |  |
| qMdSDH2 | F:ACACCATCAAGATCCTACCTTTC | Quantitative expression of MdSDH2 |
|  | R:CATTTCATGGTCTTGAGGTAGTG |  |
| qMdNINV1 | F:CTGTACCAGAGGCACCTCCGAGT | Quantitative expression of MdNINV1 |
|  | R:TTGCATGCTCTGCTCTCTCGTTG |  |
| qMdNINV2 | F:CTGTACCAGAGGCACCTCCGAGT | Quantitative expression of MdNINV2 |
|  | R:TTGCATGCTCTGCTCTCTCGTTG |  |
| qMdNINV3 | F:GGTACTTGGTAGCGAAGATGATGT | Quantitative expression of MdNINV3 |
|  | R:ACCAAACCCTTGTGCCGATTA |  |
| qMdSUSY1 | F:CTCAAGCGTGTTAAGCAACAG | Quantitative expression of MdSUSY1 |
|  | R: CTGAATGGAACACGAAGAATATC |  |
| qMdSUSY2 | F:TGTGGTTGGTGGTTACATGGATG | Quantitative expression of MdSUSY2 |
|  | R:GCTGCTATCCATCGGAACTGAC |  |
| qMdSUSY3 | F:TTATGGTTTCTGGAAGTATGTGTC | Quantitative expression of MdSUSY3 |
|  | R:GTCGATGGCTTCAGGAACAGATT |  |
| qMdSUSY4 | F:CTGTACCAGAGGCACCTCCGAGT | Quantitative expression of MdSUSY4 |
|  | R:TTGCATGCTCTGCTCTCTCGTTG |  |
| qMdFRK1 | F:CTGTACCAGAGGCACCTCCGAGT | Quantitative expression of MdFRK1 |
|  | R:TTGCATGCTCTGCTCTCTCGTTG |  |
| qMdFRK2 | F:CTGTACCAGAGGCACCTCCGAGT | Quantitative expression of MdFRK2 |
|  | R:TTGCATGCTCTGCTCTCTCGTTG |  |
| qMdFRK3 | F:AGA GTCAAGGGTATGAAGGTAGATG | Quantitative expression of MdFRK3 |
|  | R:CTCGTCCTGAAGCAAAGAAAGAT |  |
| qMdFRK4 | F:TCAGGATGAGGAGGGGCTACGAG | Quantitative expression of MdFRK4 |
|  | R:CTGCTTTAAGCACTGGAGCACAGC |  |
| qMdHxK1 | F: CTGAAAGTGGTCGGGAGCAAAC | Quantitative expression of MdHxK1 |
|  | R: TGCACGAGTGGCAACTATGTCG |  |
| qMdHxK2 | F:TGGTGGATTATACGAGCATTACA | Quantitative expression of MdHxK2 |
|  | R:TCCAGGGTATTGTGAGTGAGAG |  |
| qMdHxK3 | F:AGATTGTGGCGGATGTATGTGAC | Quantitative expression of MdHxK3 |
|  | R:CAACAGTCCTCTTGCCAAAAATG |  |
| qMdHxK6 | F:GTGGGGCAGAGTGTTTGGTGTT | Quantitative expression of MdHxK6 |
|  | R:AACCACCGTCAGAGGCCAAACC |  |
| qMdSPS1 | F:AGTGTAGTACTCAAGGGAGTTGG | Quantitative expression of MdSPS1 |
|  | R:TGCTCATGGGGAAGGCTTTAC |  |
| qMdSPS6 | F:AGGTTCTGTTGAGTATGGCAGTGAG | Quantitative expression of MdSPS6 |
|  | R:GTGCTTCAAGTGCCGCTGAGA |  |
| MdActin | F:GGACAGCGAGGACATTCAGC | Real-time PCR of actin as a reference gene in apple plants |
|  | R:CTGACCCATTCCAACCATAACA |  |


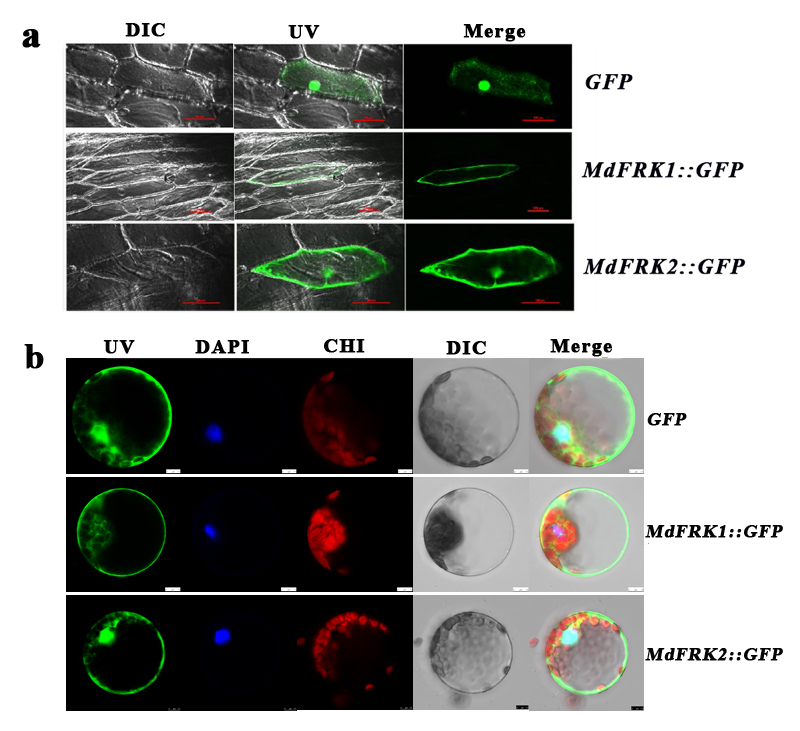


**Supplementary Figure 1** Subcellular localization of *MdFRK1-*GFP and *MdFRK2-*GFP fusion protein in onion epidermal cells (a) and *Arabidopsis* mesophyll protoplasts (b). UV: ultraviolet field; DAPI: nucleus marker; CHI: chloroplasts autofluorescence; DIC: bright field. The scale bars at the bottom represent 7.5 μm.


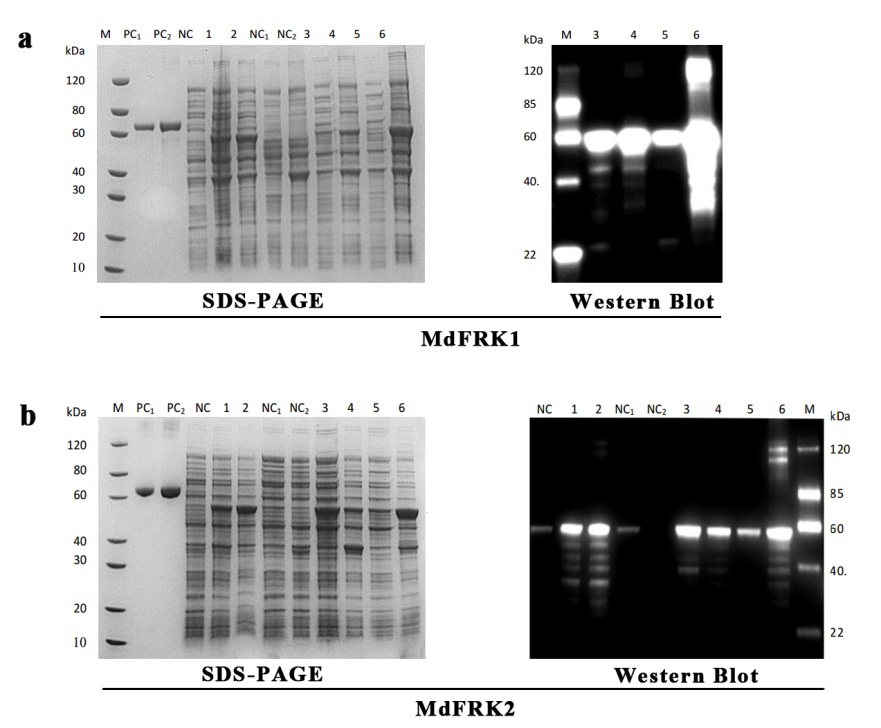


**Supplementary Figure 2** Expression analysis of *MdFRK1* and *MdFRK2* using pSUMO-M vector. (a, b) Expression products of pSUMO-M-FRK1 and pSUMO-M-FRK2. Lanes: M, protein marker; PC1, BSA (1 µg); PC2, BSA (2 µg); NC, all crude extract of recombinant *E.coli* without IPTG induction; 1, all crude extract after IPTG induction at 15°C for 16 h; 2, all crude extract after IPTG induction at 37°C for 4 h; NC_1_, supernatant of crude extract from recombinant *E. coli* without IPTG induction; NC_2_, sediment of crude extract without IPTG induction; 3 and 4, supernatant and sediment of crude extract, respectively, after IPTG induction at 15°C for16 h; 5 and 6, supernatant and sediment of crude extract, respectively, after IPTG induction at 37°C for 4 h. Western blots for both MdFRK1 and MdFRK2 used His antibodies.


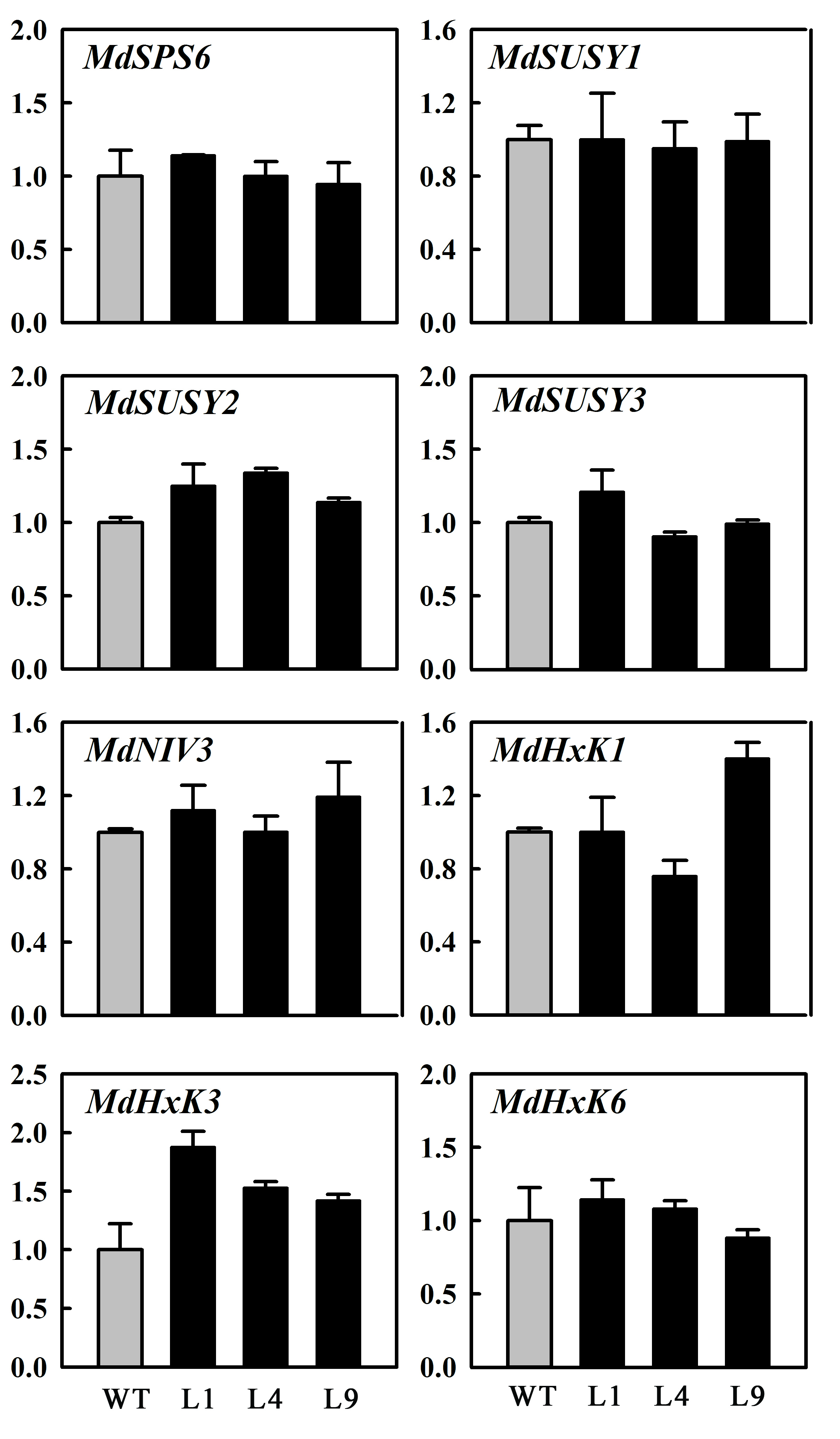


**Supplementary Figure 3** Relative mRNA expression of genes related to sucrose and sorbitol metabolism in mature leaves of wild-type (WT) and transgenic lines (L1, L4, and L9). Quantitative RT-PCR was performed with gene-specific primers, using *MdActin* as internal control. Error bars represent SD based on 3 independent replicates.


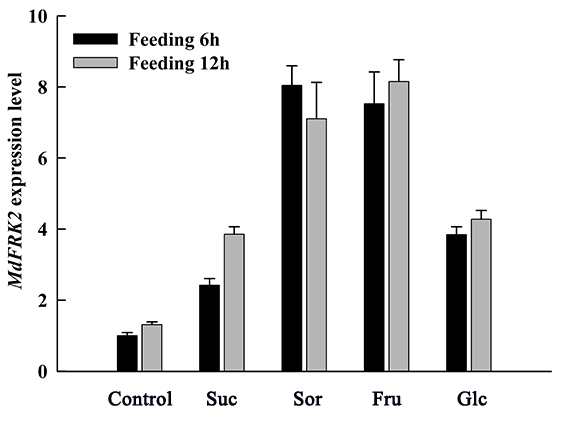


**Supplementary Figure 4** Response of *MdFRK2* expression to sugar-feeding in shoot tips of ‘Royal Gala’ apple. Two-year-old apple plants (*Malus domestica* Borkh., ‘Royal Gala’) with exogenous sugars via their transpiration streams. Actively growing shoot tips were harvested and immediately inserted into a beaker containing 200 mL of 50 mM sucrose, fructose, glucose, or sorbitol. At 6 and 12 h after feeding was initiated, samples of eight shoot tips per replicate were removed and immediately frozen in liquid nitrogen. Error bars represent SD based on 3 independent replicates.
